# Relationship Between Baseline Infarct and Clinical Outcome in Pediatric Large‐Vessel Occlusion Ischemic Stroke

**DOI:** 10.1161/SVIN.124.001357

**Published:** 2024-06-27

**Authors:** Kartik D. Bhatia, Carmen Parra‐Farinas, Elizabeth Pulcine, Nomazulu Dlamini, Ian Andrews, Sachin Gupta, Richard Webster, Christopher Troedson, Russell C. Dale, John Worthington, Kylie Tastula, Timothy Ang, Andrew Cheung, Nathan Manning, Ferdinand Miteff, Christina Miteff, Prakash Muthusami

**Affiliations:** ^1^ Children's Hospital at Westmead Clinical School University of Sydney Sydney New South Wales Australia; ^2^ Division of Paediatric Interventional Neuroradiology Hospital for Sick Children Toronto Ontario Canada; ^3^ Division of Neurology Hospital for Sick Children Toronto Ontario Canada; ^4^ Department of Neurology Sydney Children's Hospital Randwick New South Wales Australia; ^5^ T.Y. Nelson Department of Neurology and Neurosurgery Children's Hospital at Westmead Westmead New South Wales Australia; ^6^ Department of Neurology Royal Prince Alfred Hospital Camperdown New South Wales Australia; ^7^ Division of Interventional Neuroradiology Prince of Wales Hospital Randwick New South Wales Australia; ^8^ Department of Neurology John Hunter Hospital New Lambton Heights New South Wales Australia; ^9^ Department of Neurology John Hunter Children's Hospital New Lambton Heights New South Wales Australia

**Keywords:** ASPECTS, large‐vessel occlusion, mechanical thrombectomy, pediatric stroke

## Abstract

**Background:**

In adults with large‐vessel occlusion stroke, the extent of the baseline infarct measured using the Alberta Stroke Program Early Computed Tomography Score (ASPECTS) predicts outcome and is used during patient selection for mechanical thrombectomy. The relationship between ASPECTS and clinical outcome is unknown in pediatric large‐vessel occlusion stroke.

**Methods:**

Secondary analysis of a retrospective multicenter case–control study assessing mechanical thrombectomy versus medical management alone for pediatric large‐vessel occlusion stroke across 5 centers in Australia and Canada from 2011 to 2022. ASPECTS was measured on baseline computed tomography or magnetic resonance imaging–diffusion‐weighted imaging while blinded to clinical outcome and treatment status. The relationship between ASPECTS and pediatric modified Rankin scale scores at 3 months following stroke was assessed using ordinal logistic regression.

**Results:**

In total, n = 24 thrombectomy patients (F = 10, mean age, 11.3 years [SD, 4.36]) and n = 24 control patients (F = 10, mean age, 10.2 years [SD, 4.32) were included. Mean ASPECTS was 6.3 (SD, 2.37) in the thrombectomy group and 6.1 (SD, 2.64) in the control group. In patients undergoing thrombectomy, baseline ASPECTS significantly correlated with pediatric modified Rankin scale scores at 3 months (odds ratio, 1.58 [95% CI, 1.10–2.27]; *P* = 0.013). In control patients, there was no significant correlation (odds ratio, 1.17 [95% CI, 0.87–1.55]; *P* = 0.298). Six of 7 thrombectomy patients with a large baseline infarct (ASPECTS <6) had a pediatric modified Rankin scale score of 0 to 2 at final follow‐up.

**Conclusion:**

Baseline ASPECTS was significantly associated with clinical outcome in pediatric patients with large‐vessel occlusion stroke who received mechanical thrombectomy. Thrombectomy patients with low ASPECTS demonstrated favorable long‐term outcomes, suggesting ASPECTS alone should not be used to exclude pediatric patients from receiving thrombectomy.

## Graphical abstract

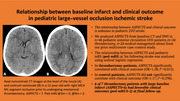

**Nonstandard Abbreviations and Acronyms**

ACA: anterior cerebral artery
ASPECTS: Alberta Stroke Program Early Computed Tomography Score
CT: computed tomography
DSA: digital subtraction angiography
ICA: internal carotid artery
LSW: last seen well
LVO: large vessel occlusion
MCA: middle cerebral artery
mRS: modified Rankin scale
NIHSS: National Institute of Health Stroke Scale
PCA: posterior cerebral artery

**Clinical Perspective**

**What Is New?**

- Baseline Alberta Stroke Program Early Computed Tomography Score significantly correlated with clinical outcome in pediatric patients with large‐vessel occlusion who underwent thrombectomy (but not in control patients), but most pediatric patients undergoing thrombectomy with large baseline infarcts (Alberta Stroke Program Early Computed Tomography Score <6) still had favorable long‐term outcomes.
- This study has demonstrated for the first time that there is a relationship between baseline infarct extent and clinical outcome in pediatric large‐vessel occlusion, but also that patients with large infarcts still did reasonably well after thrombectomy.

**What Are the Clinical Implications?**

- Alberta Stroke Program Early Computed Tomography Score can prognosticate clinical outcome in pediatric patients with large‐vessel occlusion undergoing thrombectomy, but children with large infarcts may still benefit from thrombectomy and should not be excluded from treatment on the basis of Alberta Stroke Program Early Computed Tomography Score alone.

Pediatric large‐vessel occlusion (LVO) arterial ischemic stroke has a poor natural history.^1^ Recent evidence suggests that mechanical thrombectomy is safe and effective in children,^2^ with improved clinical outcomes compared with best medical management.^3^ However, selection criteria require further clarification with variability across pediatric stroke guidelines.^4^, ^5^ The impact of baseline infarct extent, as seen on noncontrast computed tomography (CT) or magnetic resonance imaging (MRI) diffusion‐weighted imaging, upon clinical outcomes in pediatric patients with LVO stroke remains unknown.

In adults with acute LVO stroke, the extent of an established infarct on baseline CT imaging measured using the Alberta Stroke Program Early Computed Tomography Score (ASPECTS) is a strong predictor of clinical outcome.^6^ ASPECTS is an 11‐point ordinal scoring system assessing for an established infarct in 10 subregions within the relevant middle cerebral artery territory, with lower scores indicating greater infarct extent.^7^ Adult and pediatric modifications of ASPECTS using diffusion‐weighted imaging–MRI are well described.^8^, ^9^

Historically, pediatric LVO stroke was considered to have better clinical outcomes than adult LVO stroke, raising uncertainty in previous decades over the role of thrombectomy.^10^ We hypothesized that large baseline infarcts are associated with poorer outcomes in children with LVO, just as in adults. However, while modified pediatric ASPECTS correlates with outcome in pediatric stroke as a broad population,^11^ its utility for prognosis or treatment selection in pediatric patients with LVO remains unknown. In this study, we assessed the relationship between baseline infarct extent and clinical outcome in pediatric patients with acute LVO stroke.

## Methods

Because of the sensitive nature of the data collected for this study, requests to access the data set from qualified researchers trained in human subject confidentiality protocols may be sent to Sydney Children's Hospital Network at SCHN-Governance@health.nsw.gov.au.

This study was a secondary analysis of imaging and clinical data from our previously published multicenter retrospective matched case–control study (n = 52), comparing mechanical thrombectomy with medical therapy alone in pediatric patients with acute LVO stroke across 5 centers in Australia and Canada between 2011 and 2022.^3^ The methodology and protocol are previously published.^3^ Institutional ethics approval was granted at each participating site. The definition for LVO was based on the protocol of the MR‐CLEAN (Multicenter Randomized Clinical Trial of Endovascular Treatment for Acute Ischemic Stroke in the Netherlands) randomized trial for thrombectomy in adults^12^ and the Level IIa American Heart Association Guideline recommendations for thrombectomy in adult stroke,^13^ and refers to the most proximal site of intracranial occlusion inclusive of intracranial internal carotid artery, middle cerebral artery (M1–2), anterior cerebral artery (A1–2), posterior cerebral artery (P1–2), and basilar arteries. For patients with multiple sites of occlusion (eg, internal carotid artery and middle cerebral artery), the most proximal occlusion site was used for data analysis.

In summary, for the original case–control study, retrospective analysis was undertaken of pediatric stroke databases across 5 centers in New South Wales, Australia, and Ontario, Canada, across the years 2011 to 2022. All pediatric patients aged 1 month to 18 years with acute LVO stroke, confirmed on CT angiography, magnetic resonance angiography, or digital subtraction angiography, were included. Pooled data identified 31 patients who received mechanical thrombectomy and 46 patients treated with medical management alone. A hierarchal matching system (site of occlusion> age> side of occlusion> sex) was used to match patients who received thrombectomy (cases) with patients who received medical management alone (controls). Five patients who received thrombectomy with basilar artery occlusion were excluded during the matching process due to a paucity of basilar artery occlusion controls. The matching process yielded 26 patients who received thrombectomy and 26 control patients (total n = 52) for inclusion. Clinical outcomes were measured using the pediatric modified Rankin scale (ped‐mRS) score at 3 months and at final available follow‐up.

**Figure 1:**
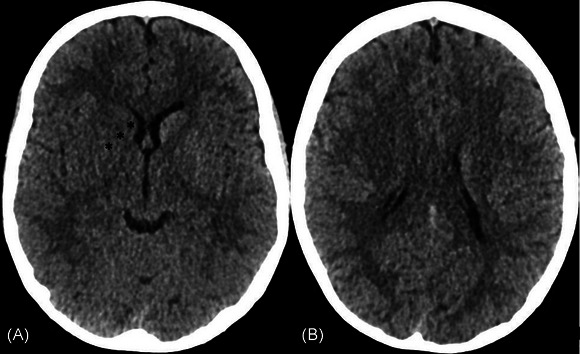
**Axial noncontrast CT images at the level of the insula (A) and centrum semiovale (B) in a 12‐year‐old with right MCA M1 segment occlusion before undergoing mechanical thrombectomy**. Established infarct is seen in the right caudate head, internal capsule, and lentiform nucleus (black asterisks). Baseline ASPECTS was 7. Pediatric mRS was 3 at 3 months and 2 at 9 months. ASPECTS indicates Alberta Stroke Program Early Computed Tomography Score; CT, computed tomography; MCA, middle cerebral artery; and mRS, modified Rankin scale.

For this secondary analysis, posterior circulation cases and controls were excluded (n = 4), as these territories are not assessed with ASPECTS. Adult protocol ASPECTS was measured using deidentified baseline noncontrast CT or MRI‐diffusion‐weighted imaging by an interventional neuroradiologist (C.P.‐F.) blinded to clinical outcome and treatment status. In patients with both baseline CT and MRI, the modality used for final treatment decision making was included. An example case is shown in Figure 1.

The relationship between ASPECTS and ped‐mRS scores at 3 months following stroke and final available follow‐up was assessed using ordinal logistic regression. Results were stratified on the basis of thrombectomy status (case versus controls).

## Results

After exclusion of posterior circulation cases (n = 4), 24 patients undergoing thrombectomy (F = 10, mean age, 11.3 [SD, 4.36] years, n = 26 thrombectomy procedures) and 24 control patients (F = 10, mean age, 10.2 [SD, 4.32] years) were included (total n = 48). Two patients undergoing thrombectomy received a second thrombectomy procedure within 72 hours due to reocclusion in the same vessel. For these, imaging acquired just before the second procedure was used for analysis.

There was no significant difference between groups for sex, age, side/site of occlusion, or imaging modality used (CT versus MRI). Baseline characteristics are detailed in Table 1. Four thrombectomy patients and 1 control patient received intravenous thrombolysis. Four patients receiving thrombectomy and 6 control patients required decompressive craniectomy. Baseline pediatric National Institutes of Health Stroke ScaleNIHSS scores at presentation were available for 18 of 24 patients undergoing thrombectomy but only 4 of 24 control patients. In the thrombectomy group, the mean baseline pediatric National Institutes of Health Stroke Scale score was 12.0 (SD = 6.15). The most common pathogenesis in each group was cardioembolic stroke (10/24 patients undergoing thrombectomy; 8/24 control patients).

**Table 1:** Baseline Characteristics and Clinical Outcomes in Pediatric Patients With Acute Anterior Circulation LVO Stroke Treated With Mechanical Thrombectomy (Cases) or Best Medical Therapy (Controls)

Variable	Cases (Thrombectomy)	Controls (Medical)	Statistical testing
**Number of patients (n)**	24	24	
**Number of thrombectomy procedures (n)**	26	0	
**Sex (n)**			χ^2^ test
Female	10	10	*p*>0.99
Male	14	14	
**Age (years)**			2‐sample t‐test
Mean	11.3	10.2	*p*=0.403
Median	13.0	11.0	
SD	4.36	4.32	
Range	3.1 – 16.0	1.9 – 17.0	
**Baseline ASPECTS**			2‐sample t‐test
Mean	6.3	6.1	*p*=0.775
SD	2.37	2.64	
Range	1 ‐ 10	0 ‐ 10	
**Location of occlusion**			χ^2^ test
Intracranial ICA	10	10	*p*>0.99
MCA: Proximal to mid M1	9	9	
MCA: M1‐2 junction to M2	5	5	
**Side of occlusion**			χ^2^ test
Left	14	13	*p*=0.771
Right	10	11	
**Stroke etiology**			N/A
Cardio‐embolic	10	8	
Dissection	3	6	
Idiopathic	4	3	
*p*aradoxical embolus	4	0	
Focal cerebral arteriopathy	0	3	
Other	3	4	
**Imaging modality used for analysis**			χ^2^ test
CT	13	10	*p*=0.386
MRI	11	14	
**IV thrombolysis administered**	4	1	
**Decompressive craniectomy**	4	6	
**Symptomatic intracranial hemorrhage**	1	1	
**Time: LSW to imaging (mins)**			Wilcoxon rank‐sum test
Mean	234.7	692.0	*p*=0.003
SD	177.9	942.2	Statistic = 40.00
Range	34–725	40–4560	
**Time: LSW to presentation (mins)**			Wilcoxon rank‐sum test
Mean	132.5	236.0	*p*=0.313
SD	139.4	363.2	Statistic = 48.00
Range	27 – 480	52 – 1641	
**Pediatric mRS at 3 months**			OR of better outcome with thrombectomy
0	5	0	OR = 4.48
1	4	1	95% 1.50‐13.44
2	7	9	*p*=0.007
3	6	7	
4	2	6	
5	0	1	
6	0	0	
**Pediatric mRS at final follow‐up**			OR of better outcome with thrombectomy
0	6	1	OR = 3.95
1	2	2	95% 1.28‐12.20
2	12	10	*p*=0.017
3	3	9	
4	0	1	
5	0	0	
6	1	1	
**Timing of final follow‐up (months)**			2‐sample t‐test
Mean	18.1	44.8	*p*<0.001
SD	15.0	28.4	
Range	3 – 48	7 – 62	

ASPECTS indicates Alberta Stroke Program Early Computed Tomography Score; CT, computed tomography; ICA, internal carotid artery; LSW, last seen well; LVO, large‐vessel occlusion; MCA, middle cerebral artery; MRI, magnetic resonance imaging; mRS, modified Rankin Scale; and OR, odds ratio.

**Figure 2:**
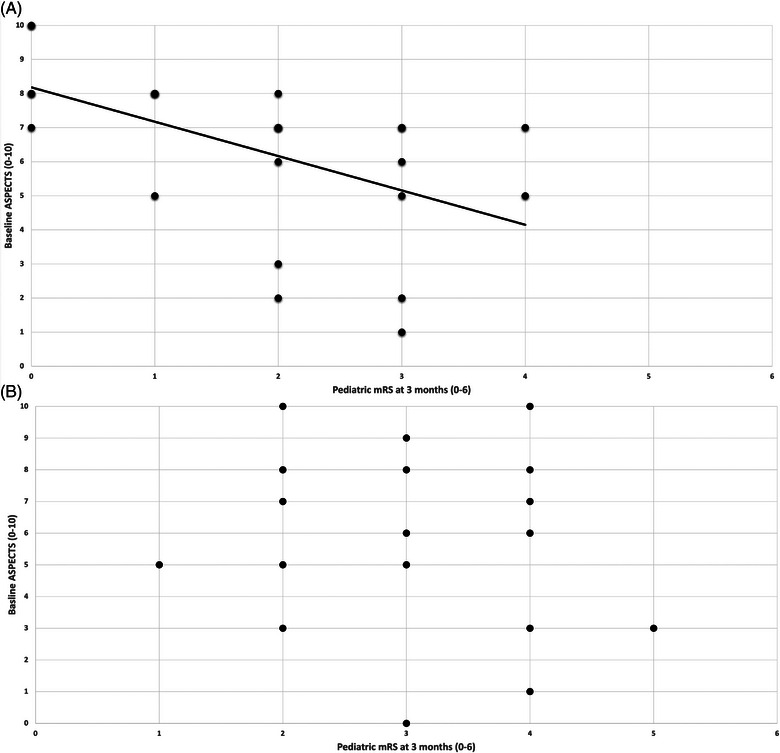
**Figure 2 (A and B): Scatter plots demonstrating the relationship between baseline ASPECTS and ped‐mRS at 3 months in pediatric acute LVO stroke patients who underwent mechanical thrombectomy (2A – Cases) or medical management alone (2B – Controls)**. ASPECTS indicates Alberta Stroke Program Early Computed Tomography Score; LVO, large‐vessel occlusion; and mRS, modified Rankin scale.

Mean ASPECTS was 6.3 (SD, 2.37) in the thrombectomy group and 6.1 (SD, 2.64) in the control group (*P* = 0.775). In patients undergoing thrombectomy, baseline ASPECTS was a significant predictor of ped‐mRS scores at 3 months (odds ratio [OR], 1.58 [95% CI, 1.10–2.27]; *P* = 0.013) and final follow‐up (mean = 18.1 months, SD = 15.0; OR, 1.49 [95% CI, 1.03–2.15]; *P* = 0.034; see Figure 2A). In control patients, there was no significant correlation at 3 months (OR, 1.17 [95% CI, 0.87–1.55; *P* = 0.298) or final follow‐up (mean = 44.8 months, SD = 28.4; OR = 1.11 [95% CI, 0.83–1.48]; *P* = 0.489). (see Figure 2B). Mixed effects model ANOVA testing showed that there was not a statistically significant interaction between treatment status and baseline ASPECTS upon ped‐mRS at 3 months (df=1.13, p=0.118).

ASPECTS was significantly associated with decompressive craniectomy status in patients undergoing thrombectomy (OR = 13.35 [95% CI, 1.52–117.11]; *P* = 0.019) but not in control patients (OR, 1.59 [95% CI, 0.31–8.11]; *P* = 0.578). In the thrombectomy group, the association between ASPECTS and clinical outcome at 3 months was more clearly demonstrated in patients with CT imaging (n = 13; OR, 2.97 [95% CI, 1.32–6.69]; *P* = 0.008) than in those who used MRI (n = 11; OR, 1.28 [95% CI, 0.80–2.06]; *P* = 0.303). There was no significant difference for time since last seen well (LSW) until angiographic imaging (CT angiography, magnetic resonance angiography) between patients receiving thrombectomy who underwent CT (n = 13; mean = 208 minutes, SD = 203.0) versus MRI (n = 11; mean = 266 minutes, SD = 145.0; *P* = 0.422). Time from LSW to imaging was significantly longer in the control group than the thrombectomy group (mean 692.0 versus 234.7 minutes; Wilcoxon rank‐sum test *P* = 0.003) despite no significant difference between groups for time since LSW to initial presentation (mean 236.0 versus 132.5 minutes; Wilcoxon rank‐sum test *P* = 0.313; see Table 1).

Seven patients undergoing thrombectomy had a large baseline infarct (ASPECTS <6), of which 3 of 7 and 6 of 7 had ped‐mRS 0 to 2 at 3 months and final follow‐up, respectively (see Table 2). A total of 9 control patients had ASPECTS <6, of which 3 of 9 and 5 of 9 had ped‐mRS 0 to 2 at 3 months and final follow‐up.

**Table 2:** Baseline Characteristics and Clinical Outcomes in Pediatric Patients With Acute Anterior Circulation LVO Stroke With Large Baseline Infarct Extent (ASPECTS 0–5)

Variable	Cases (Thrombectomy)	Controls (Medical)
**Number of patients (n)**	7	9
**Sex (n)**		
Female	5	3
Male	2	6
**Age (y)**		
Mean	10.9	9.9
SD	5.30	5.06
Range	4 ‐ 16	1.9 ‐ 16
**Baseline ASPECTS**		
Mean	3.3	3.3
Median	3.0	3.0
SD	1.7	1.9
Range	1 – 5	0 – 5
**ASPECTS**		
0	0	1
1	1	1
2	2	0
3	1	3
4	0	0
5	3	4
**Pediatric mRS at 3 mo**		
0	0	0
1	1	1
2	2	2
3	3	3
4	1	2
5	0	1
6	0	0
0 – 2 (favourable)	3	3
3 – 6 (poor)	4	6
**Pediatric mRS at final follow‐up**		
0	0	0
1	0	2
2	6	3
3	1	3
4	0	1
5	0	0
6	0	0
0 – 2 (favourable)	6	5
3 – 6 (poor)	1	4
**Timing of final follow‐up (mo)**		
Mean	20.2	31.7
SD	16.11	20.12
Range	3 ‐ 45	12 ‐ 81

ASPECTS indicates Alberta Stroke Program Early Computed Tomography Score; LVO, large‐vessel occlusion; mRS, modified Rankin Scale.

## Discussion

In this study, baseline ASPECTS was significantly associated with medium and long‐term clinical outcomes in pediatric patients with acute LVO stroke who received mechanical thrombectomy. This result supports our hypothesis and is concordant with the existing stroke literature in adults.^6^, ^14^

However, no significant association was demonstrated in the control group, despite similar baseline ASPECTS (mean 6.3 versus 6.1; *P* = 0.775). Figure 2B demonstrates control patients with favorable ASPECTS often progressed to poor clinical outcomes, likely due to completion of territorial infarction over time. Given the small samples and the overlap in CIs between the OR for controls and the OR for cases, interaction testing was undertaken using a mixed effects model ANOVA test. However, there was no significant interaction between the effects of treatment status and baseline ASPECTS upon ped‐mRS at 3 months (p=0.118).

The time from LSW to imaging was significantly longer in the control group than the thrombectomy group despite no significant time difference between groups between LSW and clinical presentation (see Table 1). This would suggest that system‐based delays in triage and imaging acquisition may have impacted upon patient eligibility and selection for thrombectomy. However, as mentioned above, there was no significant difference between groups in the extent of baseline infarct on ASPECTS.

In the thrombectomy cohort, lack of significant association between ASPECTS and clinical outcome within the MRI subgroup (n = 11) may be due to the small sample. There was no significant difference in time since last seen well until imaging between CT and MRI subgroups. If MRI is considered a more accurate representation of established infarct, this may point to the impact of thrombectomy despite infarct extent.

The correlation between baseline infarct extent and clinical outcomes in pediatric patients undergoing thrombectomy was anticipated on the basis of existing adult evidence.^14^ However, there are important differences between adult and pediatric LVO stroke pathogenesis and patient selection. Similar to adults, approximately one‐third of pediatric LVO strokes are cardio‐embolic in pathogenesis.^1^, ^3^ However, there is a greater proportion of dissection (20%), focal cerebral arteriopathy (an inflammatory arteriopathy) (10%), and idiopathic (15%) cases in the pediatric LVO population.^1^, ^3^ The decision to offer mechanical thrombectomy in such cases requires careful consideration of the potential pathogenesis based on the clinical and imaging findings. In addition, as inflammatory arteriopathies (such as focal cerebral arteriopathy) may evolve and present more slowly than embolic occlusions,^15^ the extent of baseline infarct may not necessarily predict outcomes in all cases depending on collateral artery status. As such, these results (which include patients with focal cerebral arteriopathy and dissection) will be useful to guide prognostic assessment in pediatric patients.

Patients undergoing thrombectomy with large established infarcts (ASPECTS <6; n = 7) demonstrated favorable long‐term outcomes (ped‐mRS 0 to 2 in 3of 7 and 6 of 7 at 3 months and final follow‐up). Statistical comparison with control patients with ASPECTS <6 (n = 9) was not performed given the very small samples. Recent adult randomized trials have demonstrated improved clinical outcomes with thrombectomy in patients with low ASPECTS.^16^, ^17^ Our results, in the context of recent adult evidence, suggest that ASPECTS alone should not be used to exclude pediatric patients from mechanical thrombectomy.

A pediatric modification of diffusion‐weighted imaging ASPECTS (ped‐ASPECTS) has been designed incorporating the anterior cerebral artery and posterior cerebral artery territories and using a reversed counting system to the adult versions (ie, higher scores indicate larger infarcts).^9^ Ped‐ASPECTS is designed for use with MRI only. No patients in our study had occlusion of the anterior cerebral artery or posterior cerebral artery vessels, and we had a mixture of CT and MRI. For these reasons, it was decided to use the adult ASPECTS systems.

Our study has several limitations. First, this is a secondary analysis of retrospective data and is prone to selection bias. Second, there was a mixture of CT or MRI used for final decision making, contributing to heterogeneity. Third, imaging analysis did not use automated software. Fourth, 2 patients received a second thrombectomy procedure within 72 hours of their initial procedure and the ASPECTS assessment was undertaken on imaging acquired before the second procedure; the impact of the initial stroke event on their ASPECTS may act as a confounding factor. Fifth, the number of patients undergoing thrombectomy who also received intravenous thrombolysis (n = 4) is small relative to comparable adult cohorts, forming another confounding factor impacting outcome, but is consistent with the overall low rates of patients eligible for intravenous thrombolysis in the pediatric literature. Finally, the study is limited by the small sample size and the presence of heterogeneity from patients both treated (cases) and not treated (controls) with thrombectomy.

## Conclusion

Baseline ASPECTS was significantly associated with clinical outcome in pediatric patients with LVO stroke who received thrombectomy. However, patients undergoing thrombectomy with low ASPECTS demonstrated favorable long‐term outcomes, suggesting ASPECTS alone should not be used to exclude pediatric patients from receiving thrombectomy.

## Disclosures

None.
